# Comparative Roles of IL-1, IL-6, IL-10, IL-17, IL-18, 1L-22, IL-33, and IL-37 in Various Cardiovascular Diseases With Potential Insights for Targeted Immunotherapy

**DOI:** 10.7759/cureus.42494

**Published:** 2023-07-26

**Authors:** Muhammad Abubakar, Hafiz Fahad Rasool, Izzah Javed, Saud Raza, Lucy Abang, Muhammad Moseeb Ali Hashim, Zartasha Saleem, Rai Muhammad Abdullah, Muhammad Ahmad Faraz, Khawaja Mushammar Hassan, Rakshita Ramesh Bhat

**Affiliations:** 1 Department of Internal Medicine, Ameer-Ud-Din Medical College, Lahore General Hospital, Lahore, PAK; 2 Department of Internal Medicine, Siddique Sadiq Memorial Trust Hospital, Gujranwala, PAK; 3 Department of Public Health, Nanjing Medical University School of Public Health, Nanjing, CHN; 4 Department of Biochemistry, All Saints University School of Medicine, Roseau, DMA; 5 Department of Pathology, Chughtai Institute of Pathology, Lahore, PAK; 6 Department of Emergency Medicine, The University of Lahore Teaching Hospital, Lahore, PAK; 7 Department of Anesthesia and ICU, Punjab Social Security Hospital, Lahore, PAK; 8 Department of Forensic Medicine, Post Graduate Medical Institute, Lahore General Hospital, Lahore, PAK; 9 Department of Medical Oncology, Mangalore Institute of Oncology, Mangalore, IND; 10 Department of Internal Medicine, Bangalore Medical College and Research Institute, Bangalore, IND

**Keywords:** cardiovascular disease, tocilizumab, targeted drug therapy, signaling pathways and molecules, regenerative medicine, immune system and inflammation, il-6, il-1, atherosclerosis

## Abstract

In recent years, the study of interleukins (ILs), crucial cytokines involved in immune response and inflammation, has garnered significant attention within the sphere of cardiovascular diseases (CVDs). The research has provided insights into the involvement of ILs in diverse CVDs, including arrhythmias, myocardial infarction, atherosclerosis, and heart failure (HF). ILs have emerged as promising therapeutic targets for drug interventions through their involvement in disease development and progression. This comprehensive review provides a detailed overview of ILs, elucidating their functions within the immune system and offering insights into their specific contributions to various CVDs. Moreover, the article delves into the examination of current and potential drug therapies that selectively target ILs in the management of CVDs, presenting a comprehensive analysis of the advantages and disadvantages associated with these therapeutic approaches.

A comprehensive literature review was conducted to investigate the involvement of ILs in CVDs. The relevant articles were searched on PubMed, PubMed Central, Medline, Cochrane, Google Scholar, and ScienceDirect databases. The search encompassed articles published from these databases' inception until July 12, 2023. We first examine generalized aspects of ILs, particularly CVDs. Then, we shift focus towards examining the direct impact of ILs on cardiac cells and tissue; on the immune system and inflammation; endothelial cells and vascular function; and finally, their interactions with other signaling pathways and molecules. Then, we discuss the molecular mechanisms of various ILs. Sequentially, we delve into a comprehensive analysis of the individualized role of each distinct IL in diverse CVDs, examining their specific contributions. Finally, we explore the potential for targeted drug therapy to modulate IL activity, aiming to enhance outcomes for patients burdened with CVD.

The objective is the identification of gaps in current knowledge and highlight areas that require further investigation within the context of cardiovascular medicine. Through deepening our comprehension of the intricate involvement of ILs in CVDs and harnessing their potential for targeted drug therapy, novel treatment strategies can be devised, leading to improved patient outcomes in cardiovascular medicine.

## Introduction and background

Interleukins (ILs) are a class of endogenous proteins that interact with specific receptors on cell surfaces, eliciting various cell and tissue responses and mediating communication between cells. Functioning in both autocrine (acting on the same cell that produced them) and paracrine (acting on neighboring cells) manners, these molecules belong to a smaller group of signaling proteins called cytokines, which modulate cellular behavior, including critical roles in cell growth, differentiation, and immune and inflammatory responses. They are vital in promoting immune responses, especially inflammation [1,2].

Different ILs perform distinct functions, including but not limited to B- and T-cell growth and development, activation of natural killer cells, stimulating the formation of hematopoietic cells, antibody production, immune cell proliferation, vivifying the production of interferons, mounting an inflammatory response, antigen presentation, and many more. While the role of ILs in immune system function and inflammation is well-established, their specific involvement in cardiovascular diseases (CVDs) remains an area of ongoing research.

Given the immense global burden of CVD treatment, this review aims to acquire a holistic comprehension of the underlying mechanisms linking ILs and CVDs [3]. By examining the potential impact of ILs on atherosclerosis, cardiac function, and other pathological processes, we aim to identify potential targets for therapeutic interventions. We will review the existing literature to achieve these objectives and analyze relevant studies investigating the association between ILs and CVDs. Through our research, we hope to shed light on the complex interplay between ILs and cardiovascular health, contributing to the development of novel strategies in this critical area of healthcare.

## Review

Methodology and article selection

Electronic searches were conducted on databases such as PubMed, PubMed Central, Medline, Cochrane, Google Scholar, and ScienceDirect to gather relevant articles. The search was carried out from the inception of these databases until July 12, 2023. A Boolean search approach was utilized, incorporating medical subject headings (MeSH), regular keywords, and synonyms related to the subject, including “Cardiovascular Diseases,” “Interleukins,” “Immune System,” “Inflammation,” and “Targeted molecular therapy,” both individually and in combination. The search process was optimized using the search functions provided by the websites. Articles of all types were considered.

Following the search process, 243 articles were retrieved and manually sorted. Through the initial screening of titles and abstracts, 139 articles were excluded. Additionally, grey literature was explored as part of the search process. The final reference list consisted of 78 articles that were selected based on their relevance to the topics discussed in this review.

ILs and CVDs: generalized aspects

Initially perceived as solely originating from leukocytes, it is now widely acknowledged that numerous cells in the body produce ILs. They have crucial functions in the activation and differentiation of immune cells and processes, including but not limited to proliferation, maturation, migration, and adhesion. Additionally, these proteins exhibit both anti-inflammatory as well as inflammation-promoting properties. ILs are a heterogeneous group of proteinaceous particles that regulate immune and inflammatory responses by controlling growth and differentiation. They bind to specialized cell surface receptors, eliciting various cellular and tissue responses. They can act through paracrine and autocrine signaling pathways. In addition, ILs are employed in animal studies to explore various clinical medicine-related aspects [2].

ILs have gained increasing attention recently owing to their potential significance in diverse physiological phenomena, such as immune system function, inflammation, endothelial cells (ECs), vascular function, and interaction with other signaling pathways and molecules [4]. However, further research and specific investigations are required to comprehensively understand the precise impact of ILs in these physiological processes.

Heart failure (HF) is a persistent and advancing condition marked by the heart's incapacity to adequately circulate blood to fulfill the tissues' requirements. It is a common endpoint for distinct types of cardiac injuries, including but not limited to ischemia, volume/pressure overload, and toxic-induced injuries. Moreover, it is associated with increased morbidity and death [5]. Several investigations have indicated that ILs are crucial in the inflammatory response that leads to HF development [6]. Additionally, they may also serve as prognostic indicators. For example, raised IL-1 levels in HF patients correlate with increased mortality [7]. These findings indicate that ILs substantially impact the pathogenesis and prognosis of HF.

ILs can induce the expression of cytokines that promote inflammation, attracting monocytes and neutrophils to the heart. These cells release reactive oxygen species (ROS), contributing to the oxidative burden and cardiac damage [8]. Additionally, ILs have been shown to promote cardiomyocyte hypertrophy, fibrosis, and impaired diastolic function by disrupting the extracellular matrix (ECM), all contributing to the onset and progression of HF [6,9].

During myocardial infarction (MI), ILs are produced in response to tissue injury, contributing to an acute inflammatory response. These ILs contribute to the mobilization of inflammatory leukocytes to the injured site, further exacerbating tissue damage and the progression of MI. Through specific investigations, the role of ILs in promoting inflammatory cascades and tissue remodeling after MI has been revealed [6]. Additionally, select ILs have been shown to promote clot formation, culminating in coronary artery occlusion [10]. Understanding the precise mechanisms through which ILs contribute to tissue damage during MI remains a focal point of ongoing research.

ILs have been identified as crucial contributors to the development of atherosclerosis [11]. For instance, the beta subunit of IL-1 (IL-1β) is pivotal in the preliminary stages of atherosclerotic plaque by promoting the formation of atherogenic lesions. It achieves this by upregulating ICAM-1 and related adhesion molecules, which helps monocytes adhere to endothelium. Furthermore, it stimulates ECs to produce chemokines, attracting T-cells and monocytes to the arterial wall [12,13]. There, monocytes become macrophages, engulf modified lipoproteins, and release inflammation-promoting growth factors and cytokines, forming foam cells and developing atherosclerotic plaques [13,14]. Furthermore, ILs' ability to stimulate vascular smooth muscle cell growth contributes to fibrosis within the arterial wall [15].

Direct effects of ILs

A profound comprehension of the broad implications of ILs in CVDs sets the stage for exploring their direct effects on cardiac cells and tissues.

Direct Effects of ILs on Cardiac Cells and Tissues

ILs exert direct effects on cardiac cells and tissue; among them, IL-6 stands out for its significant contribution to developing several CVDs, including HF [16]. IL-6 can stimulate cardiomyocyte hypertrophy and promote apoptosis, impairing cardiac contractile function [17]. In a recent experiment, administering IL-6 via infusion in rats exhibited a notable induction of cardiomyocyte hypertrophy and substantial fibrotic changes within the myocardial tissue [18]. Similarly, investigations have explored the involvement of IL-18 in cardiomyocyte hypertrophy regulation, myocardial contractility dysfunction, and remodeling of the ECM. However, its role in promoting apoptosis still needs to be established [19]. Furthermore, IL-1β can induce myocardial inflammation and fibrosis, leading to HF [7].

ILs as Direct Modulators of the Immune System and Inflammation

ILs, as vital components of the immune system, have a major influence on the inflammatory response, which are vital contributors to the development and progression of CVDs [20]. Inflammation-promoting cytokines like IL-1 and IL-6 promote the production of other functionally similar cytokines, leading to a cascade of immune responses and inflammation. For example, IL-1 is involved in developing atherosclerosis and can promote the production of other inflammation-promoting cytokines, including IL-6 [21]. IL-17A is another inflammation-promoting cytokine linked to atherosclerosis and CVD pathogenesis. It promotes the mobilization of inflammatory leukocytes to the luminal wall, leading to the formation of plaques, ROS generation, and subsequent oxidative cardiac damage [22,23].

Direct Effects of ILs on the Endothelial and Vascular Function

ILs strongly impact the endothelial and vascular function, integral cardiovascular system components. They achieve this through various mechanisms [24]. For example, ILs can induce endothelial dysfunction through the generation of ROS and reduce nitric oxide formation, impairing endothelium-dependent vasodilation [25,26]. Moreover, ILs can promote the proliferation and mobilization of smooth muscle cells towards the vessel, leading to plaque development and vascular remodeling [27,28]. Additionally, ILs have been found to induce angiogenesis in various cardiac conditions, including HF and MI. They can induce macrophages into a phenotype that stimulates angiogenesis, facilitating the repair of existing ones and indirectly impacting angiogenesis.

Through receptor binding on ECs, ILs may trigger angiogenesis [29]. Interestingly, it has been noted that individuals with acute coronary syndrome (ACS) have raised serum concentrations of IL-25 in coronary arteries. Raised IL-25 concentrations have demonstrated a direct association with the extent of coronary arterial narrowing and ACS incidence, indicating its potential as a biomarker for ACS [30]. Similarly, in this patient population, atrial fibrillation (AF) showed a significant association with raised levels of IL-6, indicating the unique impact IL-6 has on the pathophysiology of AF. Other biomarkers of inflammation, including C-reactive protein (CRP), did not show significant associations [31].

Interaction of ILs With Other Signaling Pathways, Their Molecular Mechanisms, With Potential Insights for Targeted Drug Therapy

ILs interact with other signaling pathways and molecules, further modulating their effects on the cardiovascular system. One example is the association between IL-1 and the NLRP3 inflammasomes and caspase-1 activation processes. The involvement of caspase-1 in the development and advancement of major CVD is intricate and multifaceted, with both positive and negative effects. It can induce the maturation of immune cells and regulate inflammation-induced apoptosis, also known as pyroptosis, through gasdermin-D activation. Thus, while caspase-1 may have a significant impact in preventing CVDs, it should be acknowledged that it can also modify the specific gene expression (e.g., NF-Kβ, SIRT-1), which can interfere with normal cellular functions and contribute to the occurrence and advancement of diverse CVDs [32].

Efforts are being made to develop inflammasome inhibitors that target NLRP3 ATPase activity (e.g., dapansutrile) or NLRP3 oligomerization. Additionally, colchicine has shown inhibitory effects on NLRP3 activation through the blockade of the specific membrane receptor openings, subsequently polymerizing the inflammasome domain [33]. Furthermore, IL-1 has been implicated in the p38, mitogen-activated protein kinase, and c-Jun N-terminal kinase (p38-MAPK-JNK) pathway activation, which is critical in acute and chronic inflammation across various diseases [11].

IL-6 has emerged as a key player in several CVDs via the axis of IL-6-gp130. Activation of the IL-6-gp130 axis initiates downstream signaling cascades, including the MAPK-ERK pathway and the Janus kinase signal transducers and activators of the transcription (JAK-STAT) pathway [34,35]. It is well-established that the action of the IL-6 primarily occurs through activating the JAK-STAT pathway alongside other intracellular signaling pathways [36,37]. These cytokines predominantly elicit STAT3 activation through a common receptor subunit, while STAT1 activation occurs to a lesser extent. ILs indirectly activate STAT proteins by activating the MAPK pathway. These STAT proteins have demonstrated their ability to induce gene transcription associated with conditions like ischemia/reperfusion (I/R) syndrome, pressure-induced concentric hypertrophy, cardioprotection, angiogenesis, cell survival, and apoptosis [38]. A study aimed at understanding how STAT3 contributes to the up-regulation of collagen genes coupled with IL-6 induction demonstrated that inhibiting STAT3 not only reversed pressure-induced concentric hypertrophy but also alleviated fibrotic tissue formation. These findings emphasize STAT3 as a promising treatment approach to modulate fibrotic tissue formation in pressure-induced concentric hypertrophy, potentially enhancing cardiac function [39]. IL-6, moreover, activates the NF-κB pathway, which regulates various genes involved in inflammation and immunity [40,41]. However, the intricate details of the molecular mechanisms of ILs in immunity and disease are beyond the scope of this review article.

CVDs, such as atherosclerosis, hypertension, cardiac fibrosis, and cardiomyopathy, are all linked to IL-6 [42]. Timing plays a pivotal role in the intricate modulation of IL-6 expression in the myocardium following an episode of MI. When IL-6 signaling is activated briefly, it can protect heart tissue, preserving it after acute damage. However, prolonged IL-6 signaling, as well as an aberrant increase in the receptor of IL-6 (IL-6R) expression, can lead to CVD, indicating that the temporal dynamics of the host response are crucial in determining whether IL-6 has a beneficial or detrimental effect on the heart [43].

The involvement of ILs in cardiomyocyte apoptosis highlights their crucial role in modulating the delicate balance between cell survival and death in the heart. Additionally, they contribute to the reduction of myocardial contractility and myocardial/vascular remodeling. Such processes contribute to the mobilization of inflammatory leukocytes in damaged myocardial tissues and vessel walls [44,45]. Cardiomyocyte apoptosis, or the programmed cell death of heart muscle cells, is a sophisticated and intricate process influenced by a multitude of cytokines and mediators [46]. Table 1 summarizes the comparative roles ILs play and their molecular mechanisms in CVDs.

**Table 1:** The role of interleukins and their molecular mechanisms in cardiovascular diseases ACS: acute coronary syndrome, CVD: cardiovascular disease, HF: heart failure, ECM: extracellular matrix, IL: interleukin, NLRP3: nucleotide-binding domain, leucine-rich-containing family, pyrin domain-containing-3, MI: myocardial infarction, SICM: sepsis-induced cardiomyopathy.

Interleukins	Role in cardiovascular diseases	References
IL-1	Inflammation-promoting cytokine involved in atherosclerosis development; linked with NLRP3 inflammasomes and caspase-1 activation processes; involved in the pathogenesis and advancement of CVDs through gene/protein expression changes; elevated levels are correlated with increased mortality in HF; and, plays a central role in many CVDs, including HF, MI, arrhythmias, pericarditis, myocarditis, and SICM.	[7,21,32]
IL-1β	Induces myocardial inflammation and fibrosis, leading to HF; involved in p38 MAPK and JNK pathway activation, which is critical in acute and chronic inflammation.	[7,11,15]
IL-6	Implicated in atherosclerosis, hypertension, aortic dissection, cardiac fibrosis, and cardiomyopathy; regulation is time-dependent and can have protective or detrimental effects on the heart depending on duration and kinetics; stimulates cardiomyocyte hypertrophy, promotes apoptosis, and impairs cardiac contractile function; and, activates multiple signaling pathways like NF-κB, PI3K-Akt, MAPK, and JAK-STAT pathways to regulate genes involved in inflammation and immunity and hence a potential target for therapeutic interventions.	[34-43]
IL-1 & IL-6 combined	Inflammation-promoting cytokines that promote the production of other inflammation-promoting cytokines, contributing to immune responses and inflammation.	[21]
IL-17A (IL-25)	Promotes recruitment of inflammatory cells to the luminal wall, leading to the development of plaque and cardiovascular events; elevated levels associated with ACS.	[22,23]
IL-18	Regulates cardiomyocyte hypertrophy, induces cardiac contractility dysfunction, and remodeling of ECM.	[19]

IL-1 and IL-6: their cardio-destructive role with potential insights for targeted drug therapy

A multitude of inflammatory leukocytes secrete IL-1, an inflammation-promoting cytokine. IL-1 comprises two different ligands, namely alpha (IL-1α) and beta (IL-1β) subunits, which have identical biological effects and use the same type 1 receptor of IL-1 (IL-1RI) to transmit signals. Binding to type 1 receptor, IL-1 receptor antagonists act as endogenous compounds which hinder the signaling of IL-1 but do not initiate signal transmission. Instead, they compete with IL-1 agonists, preventing IL-1-mediated responses. Recent findings indicate that maintaining a balance between IL-1 stimulators and inhibitors is crucial for CVDs.

The imitation and progression of various CVDs, such as atherosclerosis, HF, and MI, are significantly influenced by IL-1. Particularly noteworthy is its pivotal role in driving the genesis of atheromatous lesions and increasing inflammation within blood vessels, leading to plaque destabilization [6,47].

Moreover, studies have found that when the expression of cardiomyocyte type 2 receptor of IL-1 (IL-1R2) is induced during myocardial I/R, it not only inhibits IL-1β signaling but also effectively suppresses the induction of IL-17A ubiquitous receptor (IL-17RA). This dual effect of IL-1R2 expression attenuates IL-17A-mediated cardiac muscle death (apoptosis). Therefore, IL-1R2 overexpression in cardiomyocytes holds promising potential as an innovative therapeutic target for managing I/R syndrome. Further research on cytokine receptors in cardiomyocytes can help shed light on their intricate interplay and facilitate the formulation of more effective therapeutic modalities [48].

Increased concentrations of IL-6 in the heart have been shown to significantly contribute to MI and subsequent cardiac remodeling [49,50]. Research has shown that increased IL-6 concentration is observed in acute MI patients, reaching a peak between days one and two after the event and remaining high even after three months [51]. Furthermore, studies on animal models have revealed a notable elevation in circulating IL-6 concentrations in mice within six hours after myocardial I/R syndrome, indicating a direct correlation between elevated IL-6 concentrations and increased susceptibility to experience another MI [52,53]. These findings suggest the impact IL-6 may have on the severity of MI as well as subsequent cardiac remodeling and functional outcomes [54].

Multiple studies evidenced that elevated IL-6 concentrations in the myocardium can increase muscle mass, known as cardiac hypertrophy [55,56]. IL-6 exacerbates mitochondrial dysfunction brought about by oxidative stress via the gp130/STAT3 signaling pathway. It also increases the level of mitophagy-related proteins, thereby enhancing the removal of damaged mitochondria. However, this process generates ROS excessively, precipitating cardiomyocyte apoptosis and leading to hypertrophied myocardium and, with time, HF [57-59].

The pivotal role of IL-6 and Angiotensin II (AngII)-induced collagen deposition during cardiac hypertrophy has also been well-documented. AngII, renowned for its instrumental role in fostering cardiac hypertrophy through the renin-angiotensin-aldosterone (RAA) system, stimulates the production of IL-6. In vitro, studies have demonstrated this effect in cardiac fibroblasts as well as in cases of dysregulated pressure-induced hypertrophy, which occurs through the activation of the gp130 signaling pathway [55,60]. Nevertheless, the comprehensive comprehension of the mechanism and the role of essential signaling molecules in this collagen deposition during hypertrophy is still being determined [39].

Another mechanism by which IL-1 contributes to the development of CVDs involves its impact on the regulation of the inflammatory process and the increase in matrix metalloproteinase expression, which, in turn, can lead to cardiomyocyte hypertrophy, adverse cardiac remodeling, and a subsequent reduction in myocardial contractility [6].

IL-1α functions as an alarmin by commencing the inflammatory cascade and drawing immune cells to the site of damage or infection. It also triggers IL-1β production, which intensifies the inflammatory response even further. IL-1α concentrations are directly associated with the magnitude of the infarct in the acute phase of MI. However, during the subacute phases, IL-1β takes over as the primary cytokine responsible for cardiomyocyte death, myocardial remodeling, and the decline in contractile performance, leading to HF [33].

Role of ILs in potential targeted molecular immunotherapy

IL-1β has been a target of therapeutic interest, leading to the emergence of many IL-1 antagonists, such as recombinant IL receptor antagonists (anakinra), monoclonal antibodies (canakinumab, gevokizumab), and soluble IL decoy receptors (rilonacept). Despite these advancements, it is important to note that, at present, none of these antagonists have received an indication for use in treating CVDs [33].

The IL-1β blockade has been found to improve systolic performance in acute MI, indicating the potential of antibodies against IL-1β in protecting cardiomyocytes against I/R syndrome, as reported in recent studies [61,62]. Although they are typically well-tolerated and do not directly cause adverse effects on organs, using IL-1 antagonists can complicate pre-existing infections and has been linked to an elevated likelihood of infection-related fatalities [63].

IL-1β antagonists, including canakinumab, have undergone extensive research to explore their therapeutic potential in treating I/R syndrome. Research has indicated that canakinumab reduces major adverse cardiac events (MACE) and increases CRP levels. However, it should be noted that its usage is linked to an elevated likelihood of life-threatening infections. Conversely, colchicine has demonstrated comparable effectiveness in the prevention of MACE in ischemic heart disease patients while avoiding more cardiovascular side effects. Therefore, colchicine emerges as a favorable option for secondary cardiovascular prevention because of its cost-effectiveness, whereas canakinumab requires careful consideration due to the risk of fatal infections [64].

The effectiveness of anakinra in treating cardiovascular issues remains to be determined due to conflicting evidence. Although the drug's anti-inflammatory properties have been demonstrated in studies, its clinical benefit appears limited. On the other hand, the “CANTOS” trial has demonstrated the potential benefits of canakinumab in individuals suffering from pre-existing CVD. Nonetheless, further evidence is still warranted to confirm these findings and establish the role of canakinumab in CVD treatment. Rilonacept's effects on CVD outcomes and vascular and endothelial function have not been studied in patients with established atherosclerotic CVD [65,66].

The IL-6 blockade has been a focus of interest in targeted drug therapy for the past decade. In a clinical study, the early administration of tocilizumab, an antagonist targeting the IL-6 receptor, within a six-hour window from symptom onset in those experiencing ST-segment elevation MI demonstrated a notable improvement in the cardiac salvage index compared to the placebo, suggesting a potential protective effect. However, the final infarct size was not significantly affected. Even though tocilizumab demonstrated a significant anti-inflammatory impact on patients with a substantial inflammatory load, it did not translate into clinical benefits concerning crude cardiovascular endpoints, including mortality. The ultimate impact of tocilizumab on clinical outcomes remains uncertain [67]. Another recent study found that sarilumab, a human monoclonal antibody that interferes with IL-6 signaling by binding both soluble as well as membrane-bound receptors, is superior to adalimumab in suppressing systemic inflammation. Moreover, it has effectively reduced lipoprotein (a) levels, a known cardiovascular risk factor [68].

IL-10, IL-22, IL-33, and their cardioprotective role

IL-22 and IL-33, in comparison to cardio-destructive ILs (IL-1 and IL-6), exert a protective effect on cardiomyocytes by preventing their programed cell death caused by cardiac injury. However, the mechanisms are different. IL-22 achieves this prevention by enhancing the function of superoxide dismutase, an enzyme responsible for detoxifying ROS produced by the mitochondria. Additionally, IL-22 was shown to prevent mitochondrial membrane potential depletion, which can trigger apoptosis, inhibiting cytochrome C release, a protein also involved in the process. Similarly, IL-33's activation of ST2 signaling can prevent apoptosis of cardiomyocytes and enhance cardiac function and survival post-MI. IL-33, an innovative signaling molecule for the ST2 receptor (related to the family of IL-1 receptors), activates NF-κB and is thought to regulate apoptosis to promote cardioprotection [69,70].

Clenbuterol, an agonist that selectively targets β2-adrenergic receptors (β2AR), has been demonstrated to decrease myocardial infarct size when given prior to reperfusion in an experimental mouse model of myocardial I/R syndrome. This effect is attributed to inhibiting pro-inflammatory responses and promoting IL-10 release, an immunosuppressive IL. The study suggests that β2AR agonists, including inhalational administration, could be clinically relevant in reducing infarct size and mitigating reperfusion-induced damage in acute MI patients [71].

IL-17 and its role in vascular remodeling

Many ILs have been implicated in AngII-induced hypertension, with IL-17 standing out as a significant contributor in this regard. It establishes an intricate interplay with the RAA system and contributes to blood pressure dysregulation as well as vascular remodeling [72]. Interestingly, another IL, IL-22, has also been implicated in hypertension-related complications. Besides its involvement in vascular remodeling, IL-22 has been found to mediate the hypertensive renal injury mediated by AngII, thereby engendering renal inflammation and fibrosis, further exacerbating the hemodynamic reactivity to AngII [73]. Future research may show how to target IL-17 and IL-22 to treat these conditions effectively.

The therapeutic potential of targeting IL-17A is being explored as an attractive approach for resistant hypertension as well as for preventing target organ damage. Groundbreaking research from clinical trials has confirmed the favorable outcomes of IL-17A suppression in various inflammatory and immune disorders [74]. Once the safety profile of these drugs is established, they could be further evaluated for their potential utility in managing resistant hypertension.

IL-18, IL-37, and their role in atherosclerosis

Finally, a research study discovered a notable presence of IL-18 expression within atherosclerotic plaques of the carotid artery. The degree of IL-18 mRNA transcripts was intricately linked to clinical and pathological indications of plaque instability. The study proposed that it could be feasible to control the development and adverse outcomes of atherosclerosis by regulating IL-18 signaling, such as through the use of antagonists of IL-18 [75]. Furthermore, the recently discovered IL-37, an emerging anti-inflammatory cytokine, exhibits intriguing potential in the context of atherosclerosis. It is a natural intrinsic immunosuppressive agent with inherent abilities to protect against cardiac I/R and related diseases. IL-37, a new entity akin to the IL-1 ligand class in its genetic makeup, has been the focus of recent investigations illuminating its inhibitory capacity in dampening the inflammatory response induced by IL-18 through the MAPK inflammatory signaling pathway [76,77]. Although previous studies found some relation between IL-37 and ACS, the specific correlation and the prediction of a patient's prognosis still need to be established [78].

## Conclusions

Despite the implementation of early intervention, the widespread use of various drugs, and precise revascularization techniques, the burden of CVD remains high. ILs play a crucial role in CVDs, with IL-1α and IL-1β contributing to inflammation, cardiomyocyte death, and adverse cardiac remodeling. IL-22 and IL-33 exhibit cardioprotective effects by preventing apoptosis and enhancing cardiac function. IL-17 is associated with vascular remodeling and hypertension, suggesting its potential as a therapeutic target. IL-18 is linked to plaque instability in atherosclerosis, while IL-37 exhibits anti-inflammatory properties. Targeting these ILs holds promise for novel immunotherapies. Additional studies are warranted to ascertain safety, efficacy, and personalized interventions for improved patient outcomes.

Understanding IL-mediated mechanisms in CVDs provides valuable insights for developing targeted therapies. By harnessing the therapeutic potential of ILs, we can mitigate inflammation, promote tissue repair, and address immunological processes underlying CVD pathophysiology. Robust clinical trials are necessary to establish safety and efficacy profiles for specific IL-targeted therapies. Advancing our knowledge in this field will lead to personalized interventions and improved prognoses for patients with CVDs.
